# A New Anthracene Derivative from Marine *Streptomyces* sp. W007 Exhibiting Highly and Selectively Cytotoxic Activities

**DOI:** 10.3390/md9091502

**Published:** 2011-09-09

**Authors:** Hongyu Zhang, Hongpeng Wang, Hongli Cui, Zonggang Li, Zeping Xie, Yang Pu, Fuchao Li, Song Qin

**Affiliations:** 1 Yantai Institute of Coastal Zone Research, Chinese Academy of Sciences, Yantai 264003, China; E-Mails: hyzhang@yic.ac.cn (H.Z.); hlcui@yic.ac.cn (H.C.); zpxie@yic.ac.cn (Z.X.); ypu@yic.ac.cn (Y.P.); 2 Graduate University of Chinese Academy of Sciences, Beijing 100049, China; 3 Department of Organic and Biomolecular Chemistry, University of Göttingen, Göttingen D-37077, Germany; E-Mail: wanghongpeng@hotmail.com; 4 Yantai Institute of China Agricultural University, China Agricultural University, Yantai 264670, China; E-Mail: z_pillar@126.com; 5 Institute of Oceanology, Chinese Academy of Sciences, Qingdao 266071, China; E-Mail: lifuchao@ms.qdio.ac.cn

**Keywords:** *Streptomyces*, anthracene, structure establishment, cytotoxicity

## Abstract

A new anthracene derivative, 3-hydroxy-1-keto-3-methyl-8-methoxy-1,2,3, 4-tetrahydro-benz[α]anthracene, was isolated from the marine strain *Streptomyces* sp. W007, and its structure was established by spectroscopic analysis including mass spectra, 1D- and 2D-NMR (^1^H–^1^H COSY, HMBC, HSQC and NOESY) experiments. 3-hydroxy-1-keto-3-methyl-8-methoxy-1,2,3,4-tetrahydro-benz[α]anthracene showed cytotoxicity against human lung adenocarcinoma cell line A549.

## 1. Introduction

Natural products remain either the source or inspiration for a significant proportion of the new small-molecule chemical entities introduced as drugs [1]. Microbial natural products are an important source of both existing and new drugs [2]. Among the producers of commercially important metabolites, actinomycetes have proven to be a prolific source with a surprisingly small group of taxa accounting for the vast majority of compounds. Secondary metabolites produced by actinomycetes possess a wide range of biological activities [3–7], and the vast majority of these compounds are derived from the single genus *Streptomyce*s [8].

*Streptomyces* species are distributed widely in marine habitats and are of commercial interest due to their unique capacity to produce novel metabolites. Our group has done research on novel natural products of marine *Streptomyces* species with potential antitumor activity for 12 years [9–12]. As part of this work, in our screening of marine *Streptomyces* isolates for more active secondary metabolites, a new anthracene derivative, 3-hydroxy-1-keto-3-methyl-8-methoxy-1,2,3,4-tetrahydro-benz[α]anthracene (compound **1**) was obtained from the fermentation broth of marine *Streptomyces* sp. W007 for the first time. The structure of compound 1 was determined by LC-MS, ^1^H, ^13^C and 2D NMR spectroscopy and by comparison of the NMR data with other anthraquinone compounds, the cytotoxicities of compound **1** against human leukemic cells line HL-60, human lung adenocarcinoma cell line A549 and human hepatoma cell line BEL-7402 were studied, the details of structure and cytotoxicities of compound **1** are presented here.

## 2. Results and Discussion

### 2.1. Structure Analysis of Compound **1**

**Figure 1:**
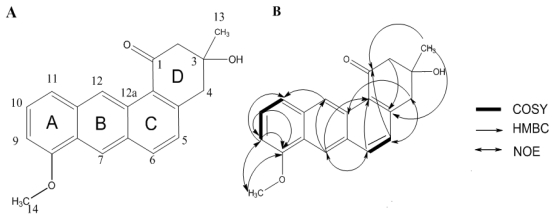
(**A**) Structure of compound **1**; and (**B**) Selected ^1^H–^1^H COSY, HMBC and NOESY correlations for compound **1**.

Compound **1** (Figure 1, Table 1) was obtained as yellow powder and showed a quasi-molecular ion [M + H]^+^ at *m/z* 307 in the LC-MS, which was consistent with the molecular formula C_20_H_18_O_3_ (calcd for 306.3551), and required 12 degrees of unsaturation. Its ^1^H NMR spectrum exhibited an aliphatic methyl singlet at δ_H_ 1.46 (3H, s), one methoxy group at δ_H_ 4.09 (3H, s), two methylene signals at δ_H_ 3.35 (2H, dd, 15.0, 20.0 Hz) and δ 2.97 (2H, dd, 10, 15 Hz) and 7 aromatic proton signals at δ_H_ 10.10 (1H, s), 8.78 (1H, s), 8.26 (1H, d, 8.6 Hz), 7.65 (1H, d, 8.25 Hz), 7.45 (1H, t, 8.25, 7.45 Hz), 7.37 (1H, d, 8.6 Hz), 6.89 (1H, d, 7.55 Hz).

The ^13^C NMR, DEPT135 and HMBC spectra of compound **1** displayed two methyl resonances at δ_C_ 28.47 (C-13, 1-Me), 55.10 (C-14), two methylenes at δ_C_ 45.41 (C-4), 54.28 (C-2), methines at δ_C_ 135.19 (C-6), 127.37 (C-5), 102.5 (C-9), 125.17 (C-12), 121.52 (C-7), 121.13 (C-11), 126.35 (C-10), quaternary carbons at δ_C_ 155.38 (C-8), 145.61 (C-4a), 134.54 (C-11a), 130.69 (C-6a), 128.74 (C-12a), 125.46 (C-12b), 124.29 (C-7a,), 70.55 (C-3), in addition to one carbonyl at δ_C_ 198.76 (C-1). By detailed comparison of the ^1^H and ^13^C NMR data of compound **1** with 1,4,8,10-tetramethoxyanthracene-2-carbaldehyde [13], the similar NMR data of the aromatic moieties confirmed that compound **1** has the anthracene skeleton. Because compound **1** required 12 degrees of unsaturation, there is a six-carbon ring next to the anthracene skeleton. In the NOESY spectrum, the proton at δ_H_ 3.35 (H-4) showed a correlation with the proton at δ_H_ 7.37 (H-5) and the proton at δ_H_ 8.26 (H-6) showed a correlation with the proton at δ_H_ 8.78 (H-7). It confirmed the combination of the B, C and D rings, also implied carbonyl group was at position 1 not position 4. The methoxyl group at δ_H_ 4.09 (H-14) showed a NOE correlation with the proton at δ_H_ 6.89 (H-9). It indicated the combination of the A and B rings.

In the COSY spectra, δ_H_ 10.10 (1H, s) and δ_H_ 8.78 (1H, s) there is no correlation with other protons, they connected with C-12 (δ_C_ 125.17), C-7 (δ_C_ 121.52), respectively. The full assignment of compound **1** was supported by the HMBC correlations. The observed long range correlations from δ_H_ 1.46 (13-Me) to δ_C_ 145.61 (C-4a) and 198.76 (C-1) in the HMBC spectrum showed that a methyl group was attached to C-3, the HMBC correlation from δ_H_ 4.06 (OH, s) to δ_C_ 28.47 (C-13, 1-Me), 45.41 (C-4), 54.28 (C-2) and 70.55 (C-3) indicated that the hydroxyl group was attached to C-3. HMBC correlation from δ_H_ 4.09 (–O–CH_3_) to δ_C_ 155.38 (C-8) indicated that the methoxy group was attached to C-8. The HMBC correlation from δ_H_ 3.35 (H-4), 7.37 (H-5) and 2.97 (H-2) to δ_C_ 198.76 (C-1) indicated that a carbonyl unit was in C-1. As shown in Figure 1B, the linkage of the Six-carbon ring with the anthracene skeleton was established by the correlations from δ_H_ 3.35 (H-4) and 2.97 (H-2) to δ_C_ 145.61 (C-4a), δ_H_ 2.97 (H-2) to δ_C_ 125.46 (C-12b).

Consequently, the structure of compound **1** was established to be 3-hydroxy-1-keto-3-methyl-8- methoxy-1,2,3,4-tetrahydro-benz[α]anthracene (Table 1).

### 2.2. Cytotoxicity and Antifungal Activities

In the cytotoxicity test, compound **1** showed no cytotoxicity against human leukemic cells line HL-60 (Table 2), a weaker cytotoxicity against human hepatoma cell line BEL-7402 compared with adriamycin (Table 2). However, compound **1** exhibited a potent inhibitory activity against human lung adenocarcinoma cell line A549. Although compound **1** showed a weaker cytotoxicity at higher concentrations than the positive control, adriamycin, while at lower concentrations, the effect of compound 1 was found to be stronger than adriamycin (Table 2).

The results demonstrated that the compound **1** almost showed no antifungal activities against the *Monilinia fructicola* (Mf) and *Colletotrichum lagenarium* (Cl) (Table 3).

## 3. Experimental Section

### 3.1. General Experimental Procedures

NMR spectra were recorded on Bruker AVANCE III™ 500 spectrometers and TMS was used as internal standard. Column chromatography was carried out on silica gel (200–300 mesh) and Sephadex LH-20 (Amersham Biosciences, Uppsala, Sweden). LC-MS were obtained by ESI on a Thermo Fisher Scientific LCQ Fleet mass spectrometer. All reagents were of analytic grade.

Precoated silica gel plates (F-254, 0.2 mm) were used for analytical TLC. HPLC was performed on a JASCO PU-2087 HPLC apparatus with a Zorbax Eclipse XDB-C18 column (250 × 9.4 mm, 5 μm; 2 mL/min; 254 nm; Agilent, Palo Alto, CA, USA).

### 3.2. Strain and Medium for Isolation and Fermentation

Marine *Streptomyces* sp. W007 isolated on Gause’s synthetic agar containing 50% natural seawater was from the marine sediments of Kiaochow Bay, Qingdao. Marine *Streptomyces* sp. W007 was characterized according to the 16S rRNA gene sequence (Accession No. JN180126 in GenBank). *Monilinia fructicola* (Mf) and *Colletotrichum lagenarium* (Cl) were from Qingdao Agricultural University (Shandong, China).

Gause’s synthetic agar (for separation of streptomycete): soluble starch 20 g, KNO_3_ 1 g, K_2_HPO_4_ 0.5 g, MgSO_4_·7H_2_O 0.5 g, FeSO_4_·7H_2_O 0.01 g, K_2_Cr_2_O_7_ 0.3 g, seawater 500 mL, deionized water 500 mL, pH 7.4. M2+ medium (for fermentation): malt extract 10 g, yeast extract 4 g, anhydrous glucose 4 g, deionized water 500 mL, seawater 500 mL, pH 7.8.

### 3.3. Fermentation

Well-grown agar cultures of *Streptomyces* sp. W007 were served to inoculate 1 L-Erlenmeyer flasks each containing 300 mL M2+ medium. The liquid medium of *Streptomyces* sp. W007 were incubated for 2 days with 180 rpm at 28 °C, then they were used to inoculate 100 Erlenmeyer flasks each containing 300 mL M2+ medium, inoculum size was 20% (v/v). At last, 100 Erlenmeyer flasks were held at 28 °C for 4 days (pH 7.8, 220 rpm).

### 3.4. Extraction and Separation

The culture broth (30 L) was filtered to give the mycelium and culture filtrate, and the filtrate was extracted by ethyl acetate. The mycelium was dried at 45 °C, and ultrasonically extracted three times by ethyl acetate and acetone respectively (15 min/time), then they were dried at 45 °C under reduced pressure. Three parts of organic layers were combined and defatted with cyclohexane to give a crude extract (8.4 g). The defatted extract was separated with silica gel column, five fractions were obtained. Fraction 1 was further purified by Sephadex LH-20 to afford A1–A5, A5 was purified by reverse column with a stepwise gradient of methanol/water (2:8–8:2) and detected by TLC to give four fractions (B1–B4). B4 was subjected to PTLC, during this step, three fractions (C1–C3) was obtained, C3 was separated by semi-preparative HPLC (JASCO PU-2087, Zorbax Eclipse XDB-C18, 250 × 9.4 mm; 2 mL/min; 254 nm; Agilent, Palo Alto, CA, USA) eluting with CH_3_OH/H_2_O(80:20, v/v) to afford compound **1** (retention time 9.37 min, 12 mg).

### 3.5. Cytotoxicity Tests and Antifungal Activities

Cytotoxic activity was evaluated using the human leukemic cell line HL-60 by the MTT (Methyl-Thiazol-Tetrozolium) method [14] and the human lung adenocarcinoma cell line A549 and human hepatoma cell line BEL-7402 by the SRB (sulforhodamine B) method [15]. The antifungal activities of compound **1** were evaluated using the agar diffusion assay [16]. Inhibition ratio was calculated according to the formula:

$$\text{Inhibition ratio}=(1-{\text{A}}_{\text{sample}}/{\text{A}}_{\text{blank}})\times 100\%$$

A_sample_: the absorbance of the sample at 540/515;

A_blank_: the absorbance of the blank at 540/515.

In the MTT assay, the cell line was grown in RPMI-1640 supplemented with 10% FBS (Fetal bovine serum) under a humidified atmosphere of 5% CO2 and 95% air at 37 °C. Cell suspensions (200 μL) at a density of 5 × 10^4^ cells/mL were plated in 96-well microtiter plates and incubated for 24 h. The compound **1** solutions (2 μL in MeOH) at different concentrations (0, 10^−4^, 10^−5^, 10^−6^, 10^−7^, 10^−8^ M) were added to each well and further incubated for 72 h under the same conditions. MTT solution (20 μL of a 5 mg/mL solution IPMI-1640 medium) was added to each well and incubated for 4 h. RPMI-1640 medium (150 μL) containing MTT was then gently replaced by DMSO (dimethyl sulfoxide) and pipetted to dissolve any formazan crystals formed. Absorbance was then determined on a Spectra Max Plus plate reader at 540 nm.

In the SRB assay, cell suspensions (200 μL) were plated in 96-cell plates at a density of 2 × 10^5^ cells/mL. Then compound **1** solutions (2 μL in MeOH) at different concentrations (0, 10^−4^, 10^−5^, 10^−6^, 10^−7^, 10^−8^ M) were added to each well and further incubated for 24 h. Following drug exposure, the cells were fixed with 12% trichloroacetic acid and the cell layer was stained with 0.4% SRB. The absorbance of SRB solution was measured at 515 nm. Agar diffusion assay: compound **1** was dissolved in MeOH/CH_2_Cl_2_ (1:1) at concentrations of 5 μg/μL. Fifty microliters of each solution was pipetted onto a sterile filter disk, which was placed onto an appropriate agar growth medium for the respective test organism and subsequently sprayed with a suspension of the respective test organism. The disks (6 mm) were air-dried in bechtop, placed on an inoculated agar plate, and incubated at 28 °C overnight, the radius of the zone of inhibition was measured in mm.

## 4. Conclusions

The results of cytotoxic tests suggested that cytotoxicities of compound **1** are selective to the tested cell lines. Adriamycin plays an antitumor function: the interaction of adriamycin and type II DNA topoisomerase causes the DNA to break. Compound **1** showed a stronger cytotoxicity at low concentration, and weaker cytotoxicity at high concentration, than positive control adriamycin; such a phenomenon implied that antitumor mechanisms of adriamycin and compound **1** were different. Possible mechanisms for cytotoxic activity against human lung adenocarcinoma cell line A549 of compound **1** are as follows: compound **1** selectively induces apoptosis in human lung adenocarcinoma cell line A549 while sparing other cells; Compound **1** has negative effect on cancer cell proliferation and the cell cycle, maybe as an enzyme inhibitor; Compound **1** can inhibit metabolism of tumor cell lines and kill them.

Therefore, further studies on the bioactivity of compound **1** are being carried out and the results described in this study suggest that compound **1** could potentially lead to antitumor agents.

## Supporting Information



## Figures and Tables

**Table 1:** ^1^H and ^13^C NMR data of compound **1** in CDCl_3_ (500 MHz).

Position	d (*J* in Hz)	δ_C_
1		198.76
2	2.97 (2H, dd, 10, 15)	54.28
3		70.55
4	3.35 (2H, dd, 15.0, 20.0)	45.41
4a		145.61
5	7.37 (1H, d, 8.6)	127.37
6	8.26 (1H, d, 8.6)	135.19
6a		130.69
7	8.78 (1H, s)	121.52
7a		124.29
8		155.38
9	6.89 (1H, d, 7.55)	102.5
10	7.45 (1H, t, 8.25, 7.45)	126.35
11	7.65 (1H, d, 8.25)	121.13
11a		134.54
12	10.10 (1H, s)	125.17
12a		128.74
12b		125.46
13	1.46 (3H, s)	28.47
14	4.09 (3H, s)	55.10

**Table 2:** Cytotoxicity of compound **1** against human leukemic cells line HL-60, human hepatoma cell line BEL-7402, human lung adenocarcinoma cell line A549.

Cancer cell line	Rate of inhibition of sample (%)	Concentration (M)				
		10^−4^	10^−5^	10^−6^	10^−7^	10^−8^
human leukemic cells line HL-60	Rate of inhibition of compound **1**	0	0	0	0	0
	Rate of inhibition of adriamycin	100	88.5	89.5	88.2	0
human hepatoma cell line BEL-7402	Rate of inhibition of compound **1**	37.5	37.0	25.7	19.5	0
	Rate of inhibition of adriamycin	80.9	85.7	63.4	32.8	15.5
human lung adenocarcinoma cell line A549	Rate of inhibition of compound **1**	65.5	62.8	61.8	47.8	48.8
	Rate of inhibition of adriamycin	100	61.8	50.8	21.4	4.3

**Table 3:** Antifungal activity of compound **1**.

Microbial activities	The radius of the zone of inhibition (mm)
Blank	6
Antifungal (Mf)	8
Antifungal (Cl)	9
