# Supplementary material for: A New Anthracene Derivative from Marine Streptomyces sp. W007 Exhibiting Highly and Selectively Cytotoxic Activities
Source: Mar Drugs. 2011 Sep 9;9(9):1502–9. doi: 10.3390/md9091502 (PMC3225931; doi:10.3390/md9091502)

Supporting Information

^{13}C of Compound **1**

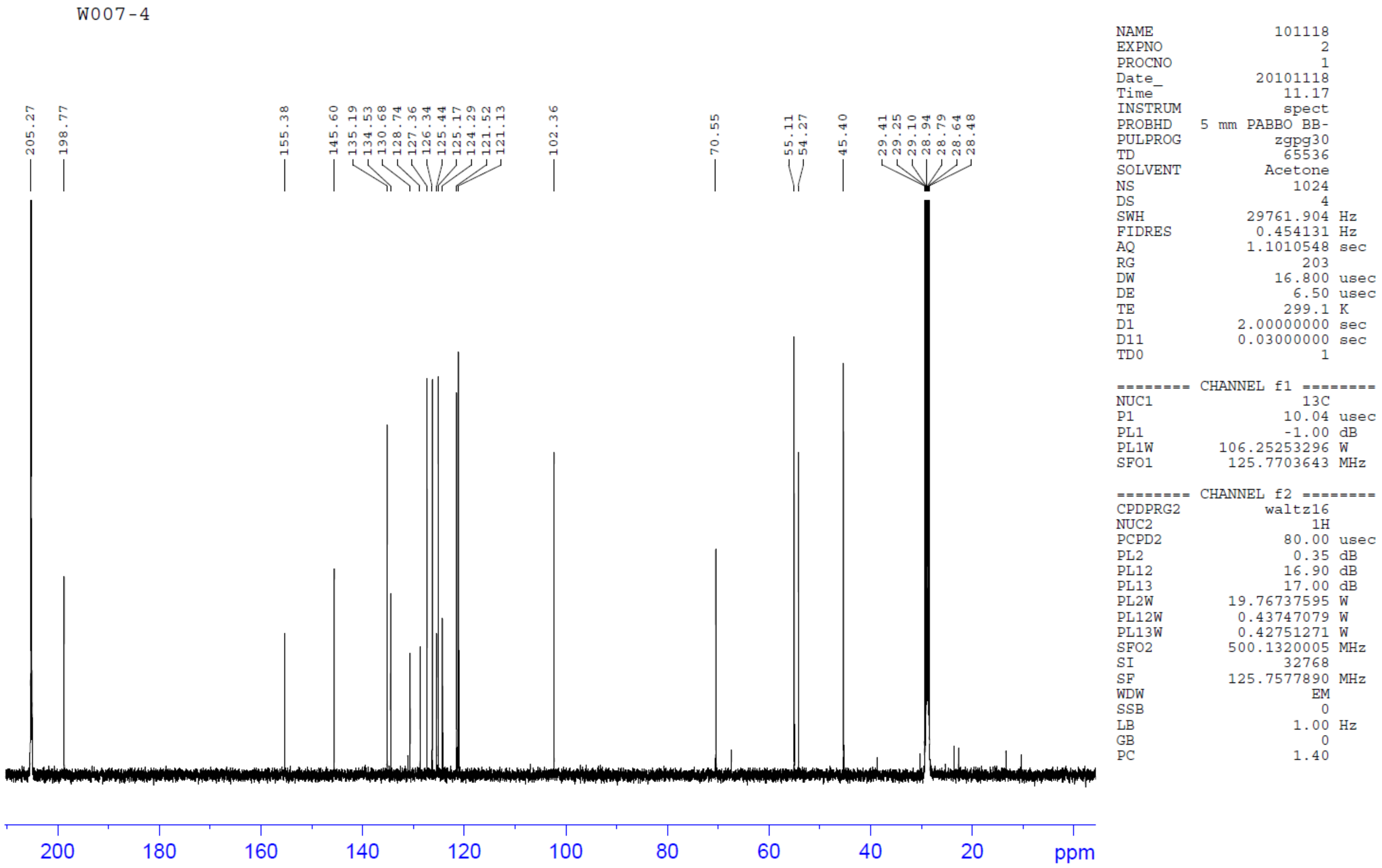

^{1}H of Compound **1**

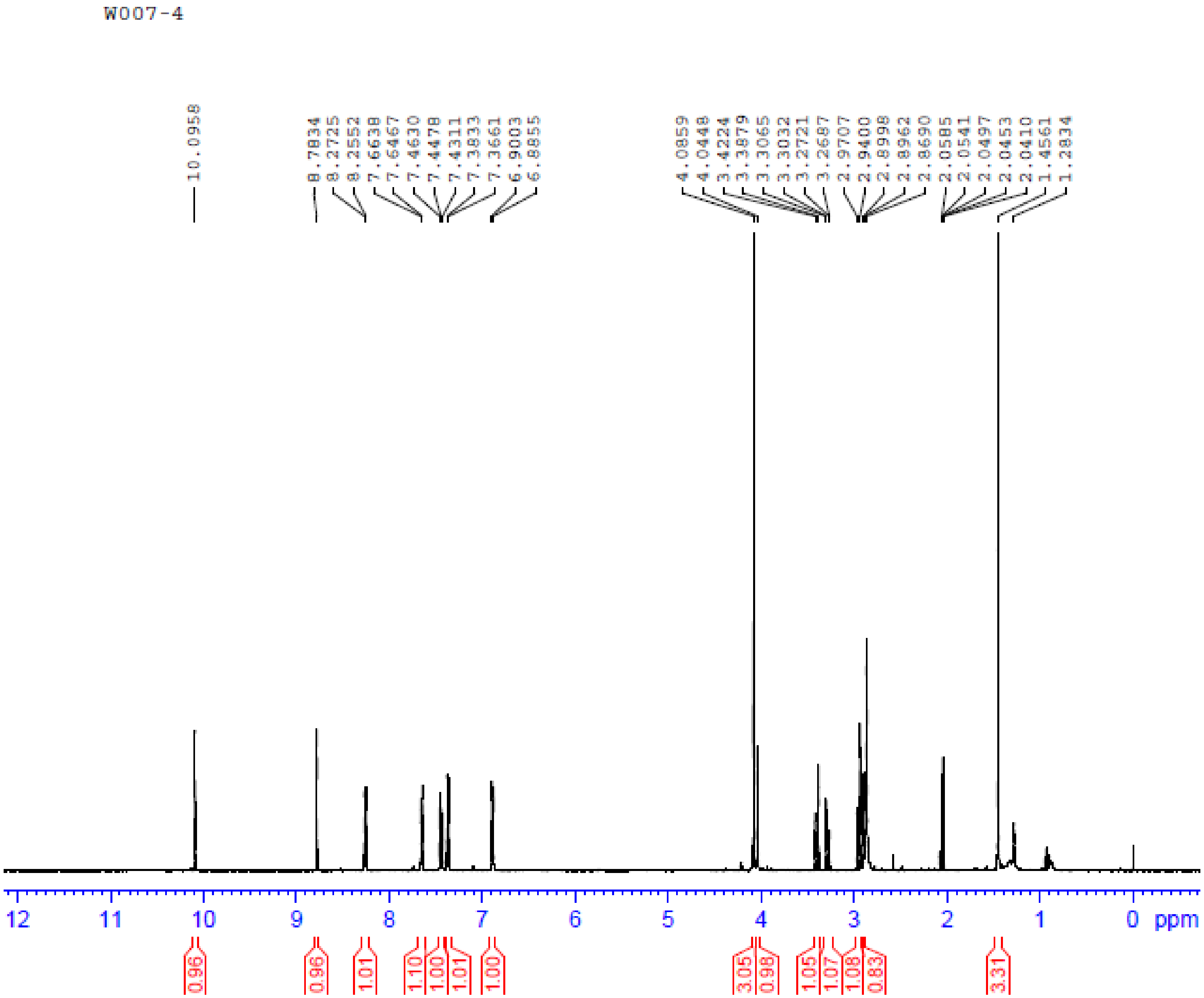

NAME	101118
EXPNO	1
PROCNO	1
Date_	20101118
Time	11.09
INSTRUM	spect
PROBHD	5 mm PABBO BB-
PULPROG	zg30
TD	65536
SOLVENT	Acetone
NS	8
DS	0
SWH	10330.578 Hz
FIDRES	0.157632 Hz
AQ	3.1719923 sec
RG	114
DW	48.400 usec
DE	6.50 usec
TE	299.0 K
D1	1.00000000 sec
TD0	1
======== CHANNEL f1 ========	
NUC1	1H
P1	11.90 usec
PL1	0.35 dB
PL1W	19.76737595 W
SFO1	500.1330885 MHz
SI	32768
SF	500.1300099 MHz
WDW	EM
SSB	0
LB	0.30 Hz
GB	0
PC	1.00

COSY of Compound **1**

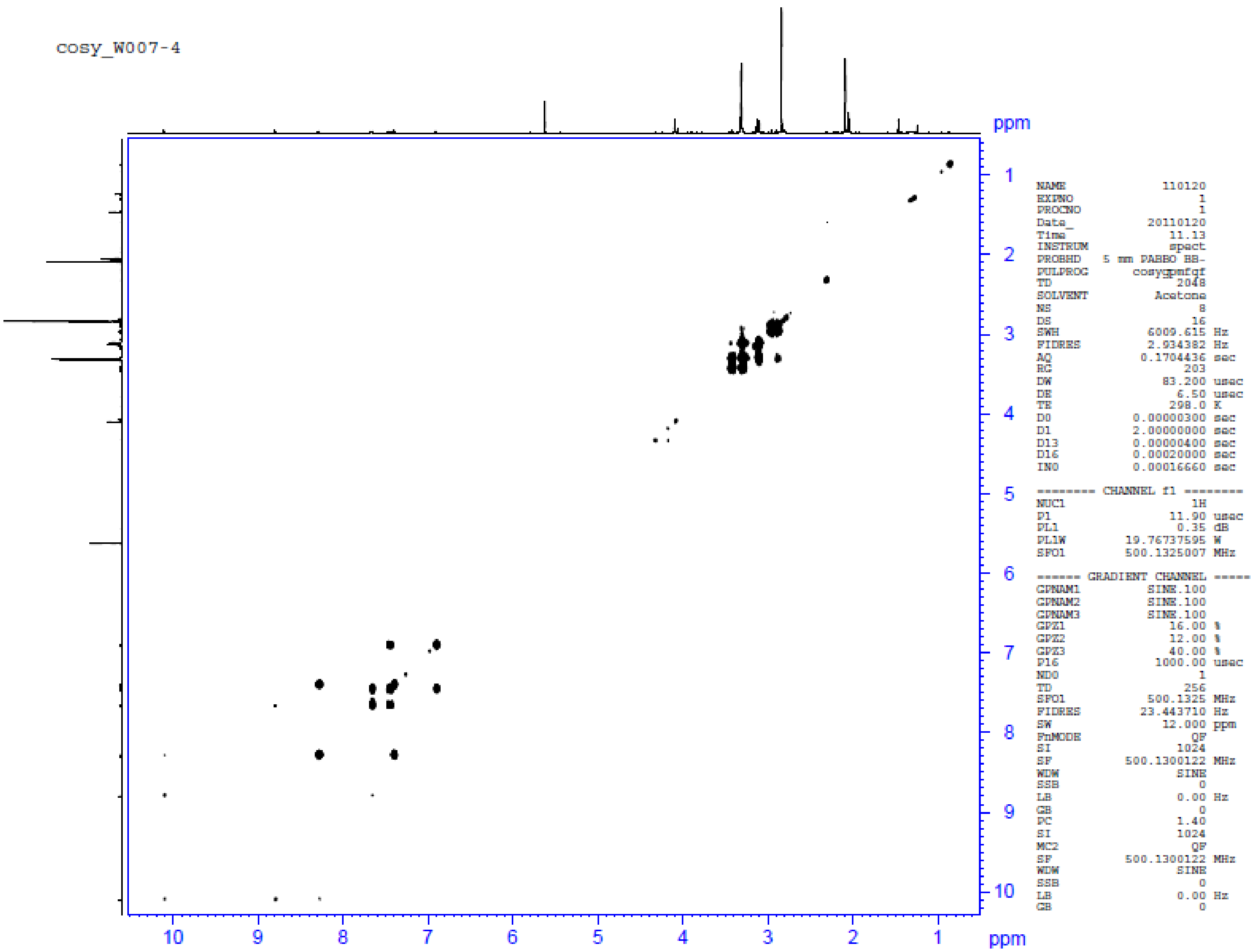

HMBC of Compound **1**

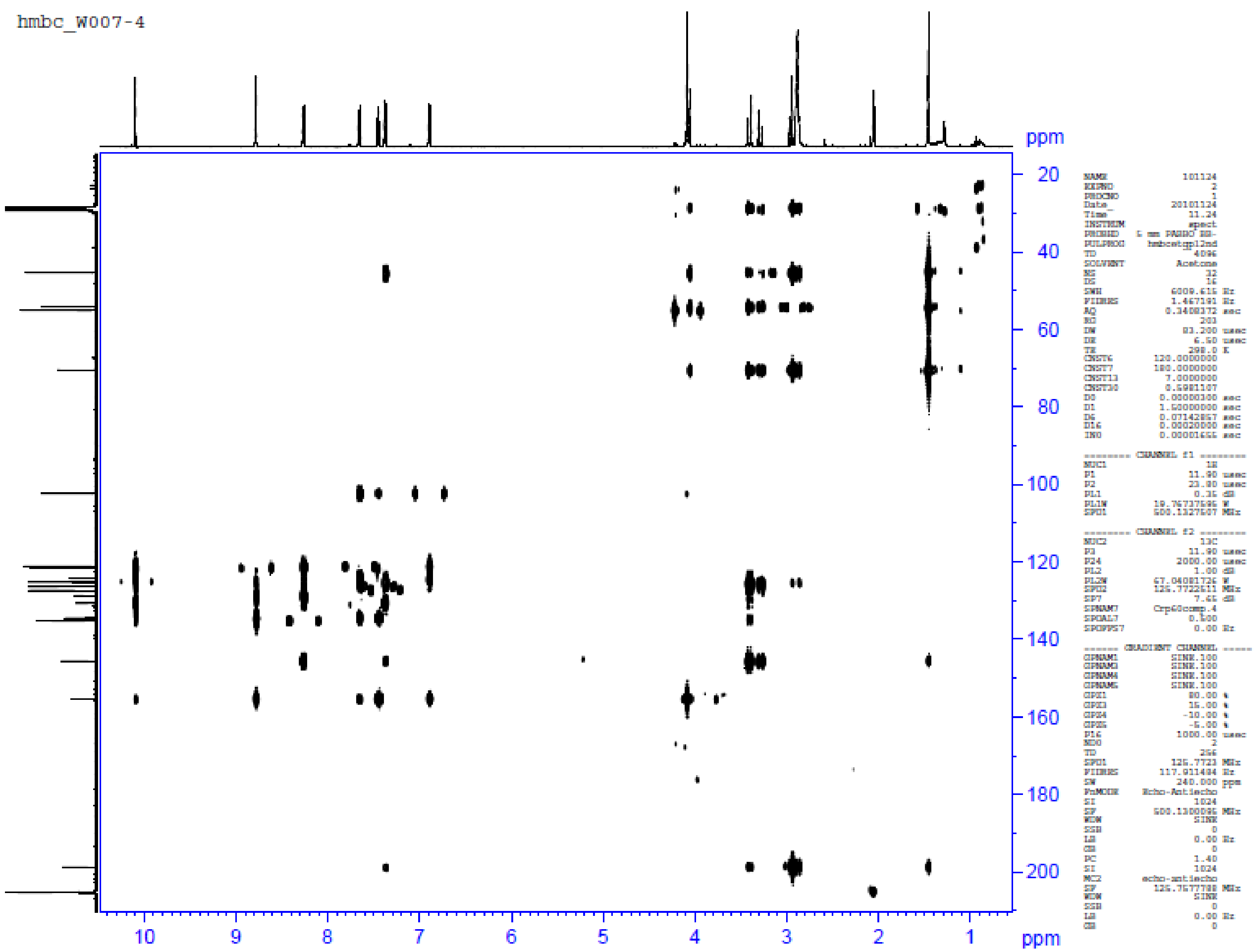

HSQC of Compound **1**

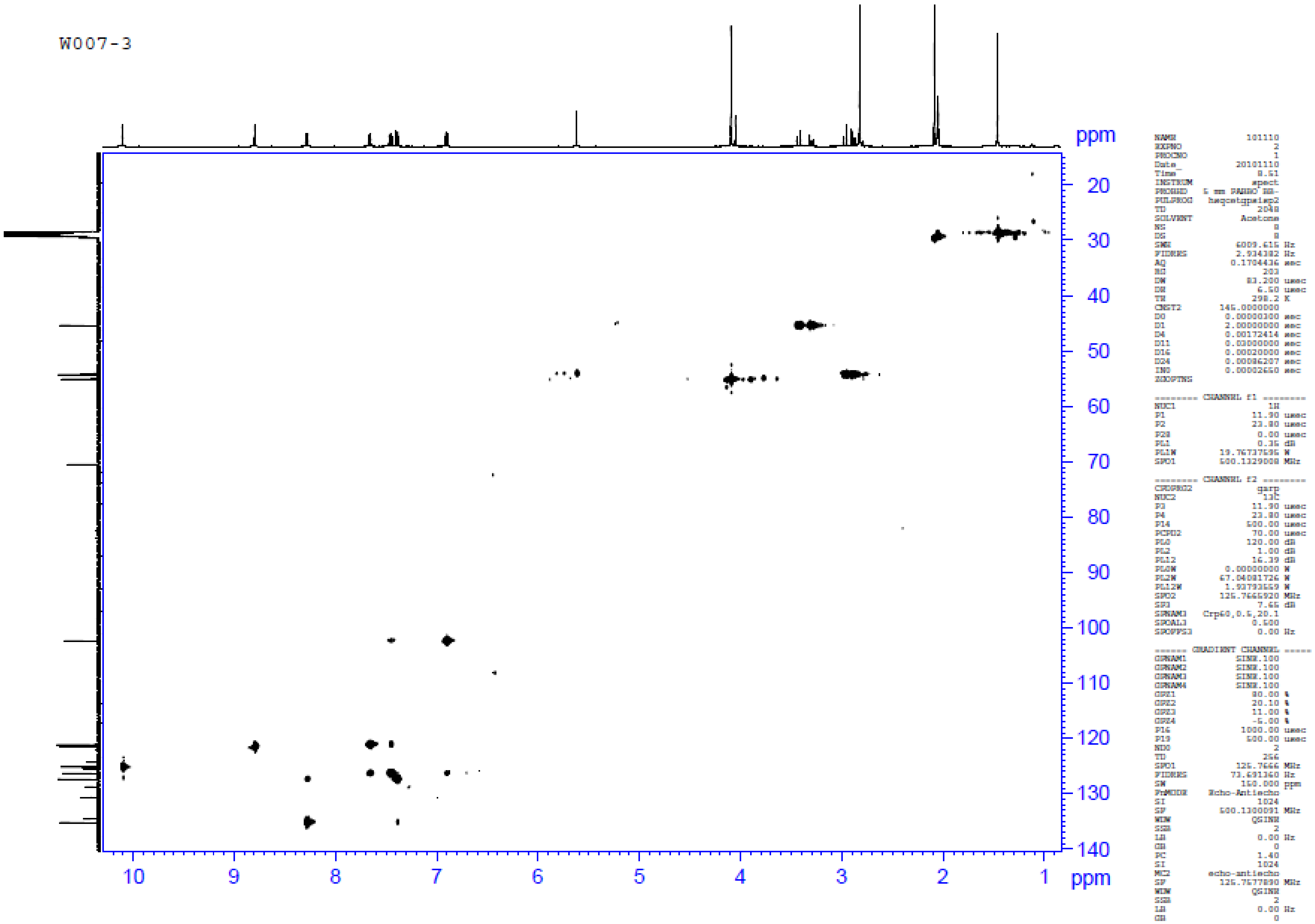

Mass Spectra of Compound **1**

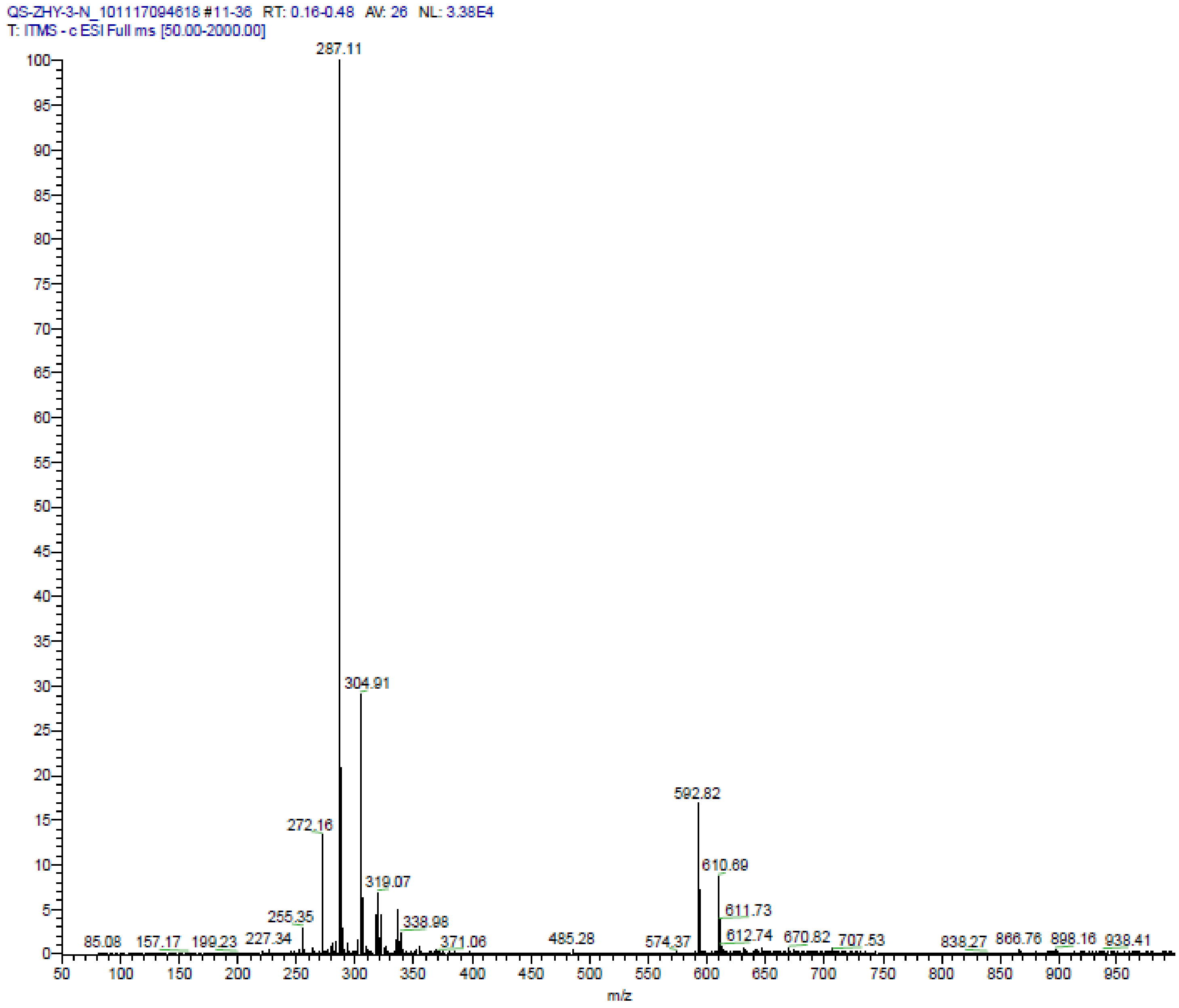

NOESY of Compound **1**

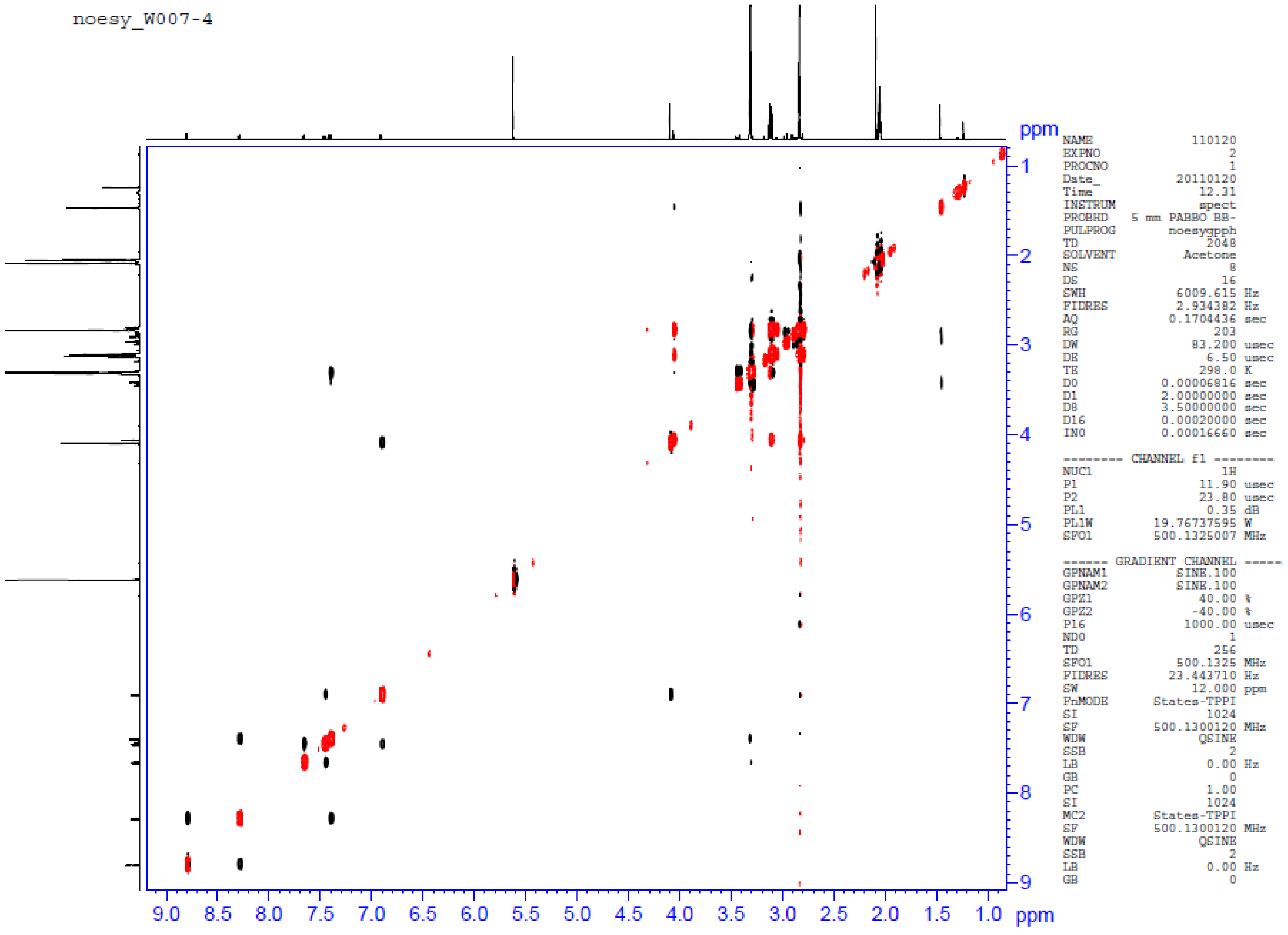

Supplement: Supplementary file 1 [file marinedrugs-09-01502-s001.pdf]
